# A cubic-quadratic phenomenological model explains the spiking, chaotic and bursting behaviors of neuron

**DOI:** 10.1038/s41598-025-98381-6

**Published:** 2025-04-25

**Authors:** Shuihan Qiu, Yeyuge Chen, Zengru Di

**Affiliations:** 1 School of Science, Jiangsu University of Science and Technology, Zhenjiang, 212100 China; 2 International Academic Center of Complex Systems, Beijing Normal University, Zhuhai, 519087 China; 3 School of Systems Science, Beijing Normal University, Beijing, 100875 China

**Keywords:** Biophysics, Neuroscience

## Abstract

In this manuscript, we present a two-dimensional phenomenological spiking neuron model. By analyzing the bifurcation diagram and phase portraits of the two-dimensional model, Andronov-Hopf bifurcation, saddle-point bifurcation and saddle-point on invariant circle bifurcation are discussed in detail. Based on the above analysis, a periodic input current is designed to simulate the basic firing mode of a single neuron. With the change of the input current frequency, the model can reproduce rich dynamical behaviors such as spike, bursts, and chaos.

## Introduction

The brain is known to reveal complex nonlinear dynamics^1–3^. In order to deeply research the working mechanism for the brain, and understand how the brain computes at neuronal description level, the research for neural systems involved in many fundamental functions of the brain has been in full swing^4–9^, such as vision, sleep, memory, emotion, odour perception, and so on. Trachtman^4^ uses data and charts to deepen our understanding of hypothalamic function. These results help clinicians better understand the pathological relationship between the visual system and the hypothalamus. In^5^, the authors gave a concise summary for some original research about the emotion regulation in memory, and indicated that a critical role for the basolateral amygdala in emotional modulation of memory.

Neurons are the basic units of brain function. Neurons are coupled to form neural circuits through synapses, and all kinds of circuits together form neural networks, which enable the complex system of the brain to emerge learning, cognition and other functions. In fact, analyzing the different behavior patterns of neurons has long been an important way to explore brain function. For example, in the neurological diseases, epilepsy, insomnia, Parkinson’s, etc., are related to the behavior pattern of neurons. A large number of researchers began to develop therapeutic regimens based on the behavior pattern of neurons, for instance Tass et al.^10,11^ investigated the therapeutic brain stimulation technique, which has proven to be a very beneficial approach to control synchronization in oscillatory networks. Unfortunately, so far there are still many psychiatric symptoms (e.g. Alzheimer’s) for which we have not fully grasped the underlying mechanism of pathology. In this regard, the multiple behavior patterns of neurons are key to focus research.

Obviously, neurons cannot fire spontaneously, but fire when they receive spikes from other neurons. To figure out what causes the spikes of neurons, mathematical modeling has become an important method to understand the dynamics of spiking models^12–23^. For instance, modeling with the Hodgkin-Huxley conductance form is one of the most common, and mathematical analysis using dynamical systems techniques to elucidate the mechanisms underlying various forms of rhythmic behavior, including spikes as well as bursts^12^. In^22^, Jia et al. proposed a biophysical neuron composed of intracellular membrane and outer membrane. This model is more suitable for recognizing neural activity in a nervous system composed of biological neurons. In^23^, the authors investigated the bidirectional-coupled neurons by means of an asymmetric electrical synapse. It is found that many states coexist, and the digital implementation of the proposed coupling neuron model is realized.

In the meantime, The research on the complex dynamical behavior of these models has also come into focus, in especial, stability^24,25^, bursting^26^, bifurcation^27–30^, synchronization^31–33^ and chaos^34–36^. Liao et al.^24^ explored the global asymptotical stability for the equilibrium for a simple delayed neural network model with three neurons based on the delay-dependent criteria and constructed Lyapunov functions. In^26^, Bao et al. developed a simplified FitzHugh-Nagumo circuit by using two anti-parallel diodes to achieve nonlinearity, and characterized the burst pattern of neurons theoretically and experimentally. Based on center manifold theory, the Hopf bifurcation problem for the FHN model model and their stability were considered in^27^. A novel chaotic hyperjerk circuit with the new phenomenon of mixed-mode bursting oscillations is given in^30^, the authors found the relatively rare and complex phenomenon of various coexisting two full Feigenbaum remerging trees. The authors in^33^ adopted the Poincaré mapping method to observe that the main reason for topological change of the synchronization mode is due to the Neimark-Sacker bifurcation. The authors derived the mathematical model that is used to examine the excitation properties of the NSRR. Analysis of the two-dimensional parameter space reveals that the NSRR exhibits periodic, chaotic patterns^36^. However, some of these models are too complex to be studied theoretically, and some are too simple to reveal rich dynamical behaviors.

Motivated by these, a Cubic-Quadratic phenomenological model is built in this study, this model is not only more intuitive in spatial geometry, but also contains only two parameters to obtain a rich phase diagram that can characterize different spiking patterns of neurons. The cubic function is responsible for the upward stroke of the spike, which stands for the effect of many factors such as capacitance, numerous ionic current, external clock on the membrane voltage. The quadratic function is responsible for repolarization of the spike (downstroke), which represents potassium channel activation.

The rest of this paper is organized as follows. In Sect.2, a simplified spiking neuron model is proposed and its stability is analyzed. In Sect.3, the bifurcation and dynamical behavior of the model are further described in detail. In Sect.4, a periodic input current is applied to the model. Conclusions are given in Sect.5.

## Preliminaries

### Model description

Some representative neural network models, in recent decades, have been put forward. In especial the Hodgkin-Huxley model, which demonstrates the simulation of action potentials under the interaction of four nonlinear equations. Although this model can simulate the activity of biological nerves very well, it is too complicated to analyze it theoretically with mathematical method.

In this paper, a reduced two-dimensional Cubic-Quadratic model are presented. The most typical spike behavior of neurons is determined by thresholds, where the voltage rises when it reaches a certain threshold and falls as it rises to a certain threshold. The voltage of a neuron varies within a specific region. In Eq. (1), $-W^{3}$ limits the voltage to a limited range, 30*W* is to promote the voltage rise which is to accelerate the occurrence of depolarization, *Z* means the factor that lowers the voltage such as the number of $K^{+}$ channels. The cubic function is responsible for the upward stroke of the spike. The term $5(W+v)^{2}$ means that the change in voltage will promote the opening of ion channels and *v* is an adjustable parameter, $-Z$ means that the open channel will close at a certain rate, The quadratic function is responsible for repolarization of the spike (downstroke). Therefore, the reduced model is always identical with the original model both qualitatively and quantitatively.

We substitute respectively *W* and *Z* for *V* (membrane voltage) and *R* (recovery variate), the reduced model is acquired:

1 $$\begin{aligned} \frac{dW}{dt}= & -W^{3}+30W-Z+E, \nonumber \\ \tau (Z)\frac{dZ}{dt}= & 5(W+v)^{2}-Z, \end{aligned}$$

where *W* stands for membrane potential, *Z* stands for recovery variable, *E* is the external input current and *v* means the ability of different kinds of cells to recover potential, which is an adjustable parameter, $\tau (Y)$ is a time constant, we set $\tau (Y)=1$ in this paper unless otherwise specified.

The two parameters *E* and *v* have very clear effects on the geometrical structures of the model. The phase plane of the reduced two-dimensional model consists of W-nullcline and Z-nullcline, where the cubic function represents W-nullcline, and the value of the parameter *E* determines whether W-nullcline moves vertically up or down, the other quadratic function denotes Z-nullcline, and the value of the parameter *v* determines whether Z-nullcline moves horizontally to the left or to the right. That is where the Cubic-Quadratic system/model (C-Q system/model) got its name.

### Stationary states and their stability

Equation (1) can produce two nullclines defined as follows

2 $$\begin{aligned} f(W,Z)= & -W^3 +30W + E - Z=0, \nonumber \\ g(W,Z)= & 5(W+v)^2 - Z=0. \end{aligned}$$

By virtue of the association between *g*(*W*, *Z*) and *f*(*W*, *Z*), the C-Q model can have 1 to 3 fixed points. Therefore, setting $f(W,Z)=g(W,Z)$, we gain the third-order polynomial for *W*:

3 $$\begin{aligned} h(W,v)=-W^{3}-5W^{2}+(30-10v)W+E-5v^{2}=0. \end{aligned}$$

where *h*(*W*, *v*) is steady-state function.

#### Impact of parameter v on stability

Evidently, as the diverse values of *E*, *v*, the C-Q model (1) will produce diverse real roots. Subsequently, We firstly fixed $E=70$ to research the impact of parameter *v*.

Based on the *h*(*W*, *v*) (3), we can acquire three equilibrium points $W_{i}, i=1,2,3$ for the parameter *v*

4 $$\begin{aligned} W_1= & P_{1}-P_{2} - 5/3, \nonumber \\ W_2= & P_{2}/2 -P_{1}/2- 5/3- (3^{1/2}(P_{1}+P_{2})i)/2, \nonumber \\ W_3= & P_{2}/2 -P_{1}/2- 5/3+ (3^{1/2}(P_{1}+P_{2})i)/2, \nonumber \\ P_{1}= & (25v/3 - 5v^2/2 + ((10v/3 - 115/9)^3 + (25v/3 - 5v^2/2 \nonumber \\&~&+ 145/27)^2)^{1/2} + 145/27)^{1/3}, \nonumber \\ P_{2}= & (10v/3 - 115/9)/(25v/3 - 5v^2/2 + ((10v/3 - 115/9)^3\nonumber \\&~&+ (25v/3 - 5v^2/2 + 145/27)^2)^{1/2} + 145/27)^{1/3}. \end{aligned}$$

**Fig. 1:**
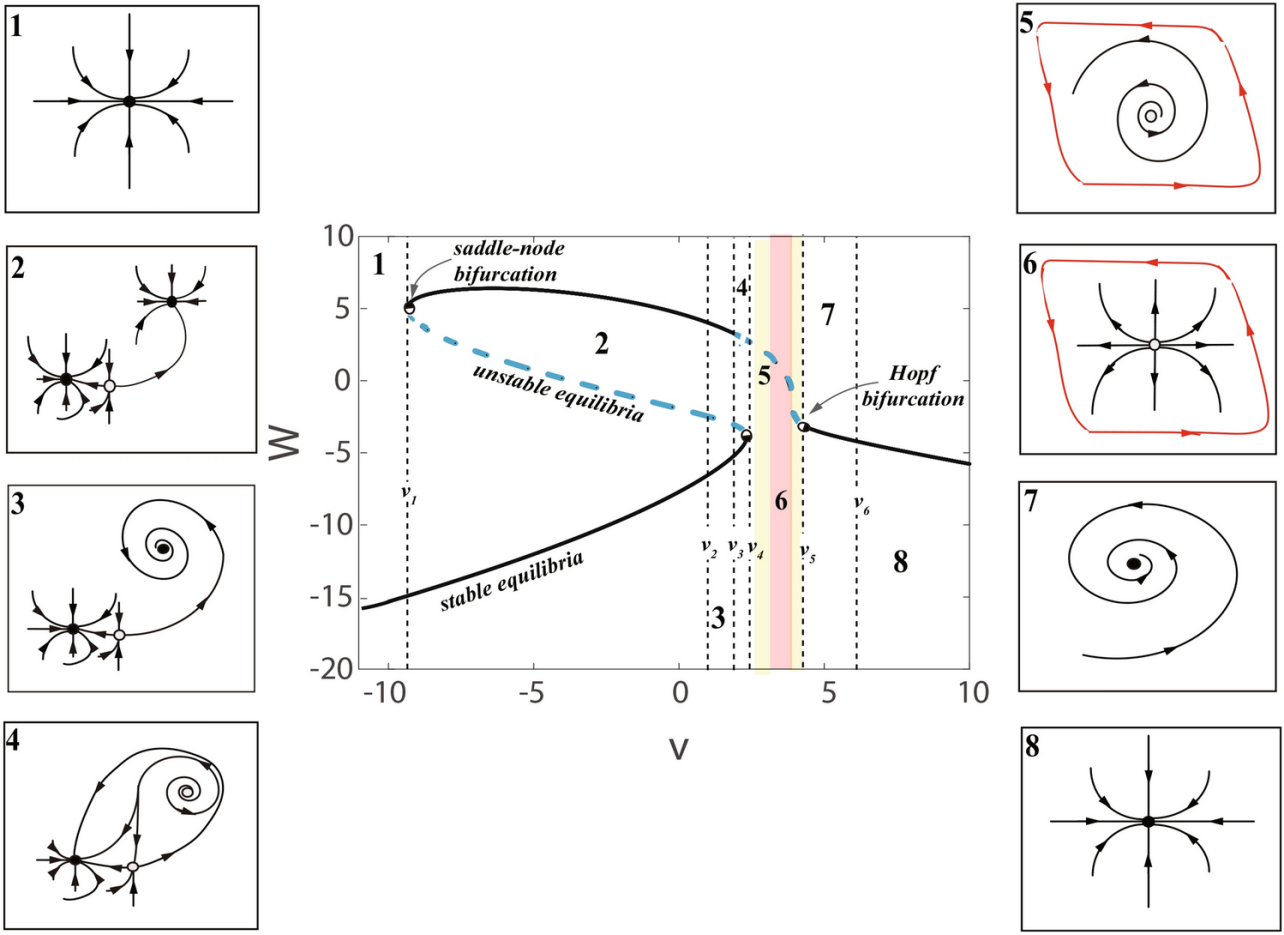
Bifurcation graph and phase portraits for the C-Q model with the parameter *v*. The black dotted lines represent $v_{1}\thickapprox -9.359, v_{2}=1.176, v_{3}=2.053, v_{4}\thickapprox 2.3304, v_{5}=4.2737, v_{6}=4.87$. The insets shows the phase portraits of the corresponding equilibrium points of each interval.

The $1\sim 3$ equilibrium points are displayed in Fig. 1, the parameter *v* must satisfy the following conditions:

1. If $v_1=-9.359>v$ or $v>v_4=2.3304$, the unique equilibrium point $P_e=(W_e,Z_e)$ is given as follows,

5 $$\begin{aligned} \left\{ \begin{array}{ccc} W_e& =& W_3, \\ Z_e& =& 5(W_3+v)^2, \end{array}\right. \end{aligned}$$

or

6 $$\begin{aligned} \left\{ \begin{array}{ccc} W_e& =& W_1, \\ Z_e& =& 5(W_1+v)^2, \end{array}\right. \end{aligned}$$

2. If $v=v_{1}\thickapprox -9.359$ or $v=v_{4}\thickapprox 2.3304$, there are two equilibrium points,

7 $$\begin{aligned} \left\{ \begin{array}{ccc} W_{e1}& =& W_3=-14.9293, \\ W_{e2}& =& W_1=W_2=4.9967, \\ Z_{e1}& =& 2.9496e+03, \\ Z_{e2}& =& 95.1483, \end{array}\right. \end{aligned}$$

or

8 $$\begin{aligned} \left\{ \begin{array}{ccc} W_{e1}& =& W_1=2.8099, \\ W_{e2}& =& W_2=W_3=-3.9049, \\ Z_{e1}& =& 132.1134, \\ Z_{e2}& =& 12.3953, \end{array}\right. \end{aligned}$$

3. If $-9.359=v_{1}<v<v_{4}= 2.3304$, three equilibrium points are acquired,

9 $$\begin{aligned} \left\{ \begin{array}{ccc} W_{ei}& =& W_i, \\ Z_{ei}& =& 5(W_i+v)^2, \\ \end{array}\right. \end{aligned}$$

where $(W_{ei},Z_{ei})$ represent the equilibrium points, $i=1,2,3$.

By the lights of Jacobi matrix $J_{v}$, we can judge the stability of $P_{ek}=(W_{ek},Z_{ek})$.

$$\begin{aligned} J_{v}=\left[ \begin{array}{ccc} -3W^{2}_{ek}+30 & ~~~~ -1\\ 10W_{ek}+10v & ~~~~ -1 \end{array}\right] . \end{aligned}$$

In Fig. 1, during $v<-9.359$, the C-Q model (1) owns one stable node (as in illustration 1). Particularly, $v_{1}\thickapprox -9.359$ as well as $v_{4}\thickapprox 2.3304$, and this is the critical point of the model where the model has a saddle point bifurcation. If the value of *v* gradually approaches $v_{4}$, that is, $v=v_{4}\thickapprox 2.3304$ (see the illustration 4), then saddle-point appear on invariant circle bifurcations. When $v\in (-9.359,2.3304)$, the model owns three equilibria, the stability for those equilibria in interval ($v_{1}, v_{2}$) is stable node, unstable saddle and stable node (top-down, as in illustration 2), in interval ($v_2, v_3$) is stable focus, unstable saddle and stable node (top-down, see the illustration 3, the system has two stable equilibria separated by an unstable equilibrium. Such a system is called bistable). In contrast with the illustration 3, the illustration 4 in interval ($v_3, v_4$) shows unstable focus, unstable saddle, stable node. (such a system might be called bi-instable when the system has two instable equilibria). In this cases the attraction domains are separated by a pair of the trajectories of unstable equilibrium, which converge to the saddle equilibrium, as shown in illustration 5. One unstable fixed point occur in interval $(v_4, v_5)$ (colored region), that are unstable focus (faint yellow, see the illustration 5) and the unstable node (light pink, see the illustration 6). After $v_{5}<v$, the equilibrium becomes a stable state with a stable focus (see the illustration 7) and a stable node (see the illustration 8). As the value of *v* gradually approaches $v_{5}=4.2737$ (critical point) from the direction greater than $v_{5}=4.2737$, once the point is passed Hopf bifurcation begins.

A quantity of numerical instances are listed to further make clear the above consequences, for the illustration 1, we consider $v=-10$, the eigenvalues for sole equilibria $P_{e}=(-15.3187,3205.2)$ are $\lambda _1=-674.3637, \lambda _2=-0.6240$. Because $\lambda _1,\lambda _2<0$ and are real value, this point is a stable node. For the illustration 2, let $v=-5$, we acquire $\lambda _1=-0.578, \lambda _2=404.5919$ for the first fixed point $P_{e1}=(-12.0301,1450.1)$, which is a stable node; We acquire $\lambda _1= 29.8102, \lambda _2=-2.3875$ for the second equilibria $P_{e2}=(0.7251,91.3739)$, which is an unstable saddle point since there are one positive and one negative real values; We acquire $\lambda _1= -89.1072, \lambda _2=-1.1481$ for the third equilibria $P_{e3}=(6.3049,8.5138)$, which is also a stable node. For the illustration 3, let $v=1.5$, considering the first equilibria $P_{e1}=(-5.8469,94.4777)$, whose eigenvalues are $\lambda _1=-0.3976, \lambda _2=-73.1611$, which is a stable node; Considering the second equilibria $P_{e2}=(-2.7745,8.1218)$, whose eigenvalues are $\lambda _1= -2.3734, \lambda _2=8.2799$, which is an unstable saddle point; Considering the third equilibria $P_{e3}=(3.6215,131.1488)$, whose eigenvalues are $\lambda = -5.1729 \pm 5.8139i$. The equilibria is a stable focus because $\lambda _1$ and $\lambda _2$ a pair of complexes conjugate numbers with negative real parts. For the illustration 4, let $v=2.1$, the eigenvalues for the first point $P_{e1}=(-4.7882,36.1321)$ are $\lambda _1= -39.4792, \lambda _2=-0.3014$. It is a stable node; The eigenvalues for the second point $P_{e2}=(-3.2722,6.8703)$ are $\lambda _1= -5.0303, \lambda _2=1.9084$. This point is an unstable saddle. The eigenvalues for the third point $P_{e3}=(3.0604,133.1486)$ are $\lambda _1= 0.4509 + 7.0355i, \lambda _2=0.4509 - 7.0355i$. It is an unstable focus because the eigenvalues are a pair of complexes conjugate numbers with positive real parts. For the illustration 5, let $v=2.35$, we get $\lambda _1=2.8456 + 6.0486i, \lambda _2=2.8456 - 6.0486i$ for a unique equilibria $P_{e}=(2.7874, 131.9644)$, which is an unstable focus. For the illustration 6, let $v=3$, the eigenvalues for the sole equilibria $P_{e}=(1.9030,120.1970)$ are $\lambda _1=16.3020, \lambda _2=1.8338$, this point is unstable node owing to $\lambda _1, \lambda _2>0$ and are real values. For the illustration 7 and 8, let’s take $v=4.5/5$ to compute the eigenvalues are $-2.0117 \pm 3.2861i$ ($P_{e}=(-3.3178,6.9880)$) and $-8.0344, -2.9200$ ($P_{e}=(-3.6494,9.1206)$), which are stable focus and node.

#### Impact of parameter E on stability

Similarly, let $v=3$ and we probe into the impact of the parameter *E*. Three equilibrium points $W_{i}, i=1,2,3$ with the parameter *E* are given:

$$\begin{aligned} W_1= & - \textrm{Z} - (\sqrt{3}(P1- P2)i)/2 - 5/3, \\ W_2= & P1 - 5/3 + P2, \\ W_3= & - \textrm{Z} + (\sqrt{3}(P1- P2)i)/2 - 5/3, \\ \textrm{Z}= & (\sqrt{((\alpha /2 + 5^3/27)^2 - 5^6/729)}- 5^3/27 - \alpha /2)^{1/3}/2,\\&~&- 5^2/(18*(\sqrt{((\alpha /2 + 5^3/27)^2 - 5^6/729)} - 5^3/27 - \alpha /2)^{1/3}), \\ P1= & (\sqrt{(- 5^6/729+(5^3/27 + \alpha /2)^2 )} - 5^3/27 - \alpha /2)^{1/3}, \\ P2= & 5^2/(9*(\sqrt{(- 5^6/729 + (\alpha /2 + 5^3/27 )^2 )} - 5^3/27 - \alpha /2)^{1/3}), \end{aligned}$$

where $$\alpha =E+45$$.

**Fig. 2:**
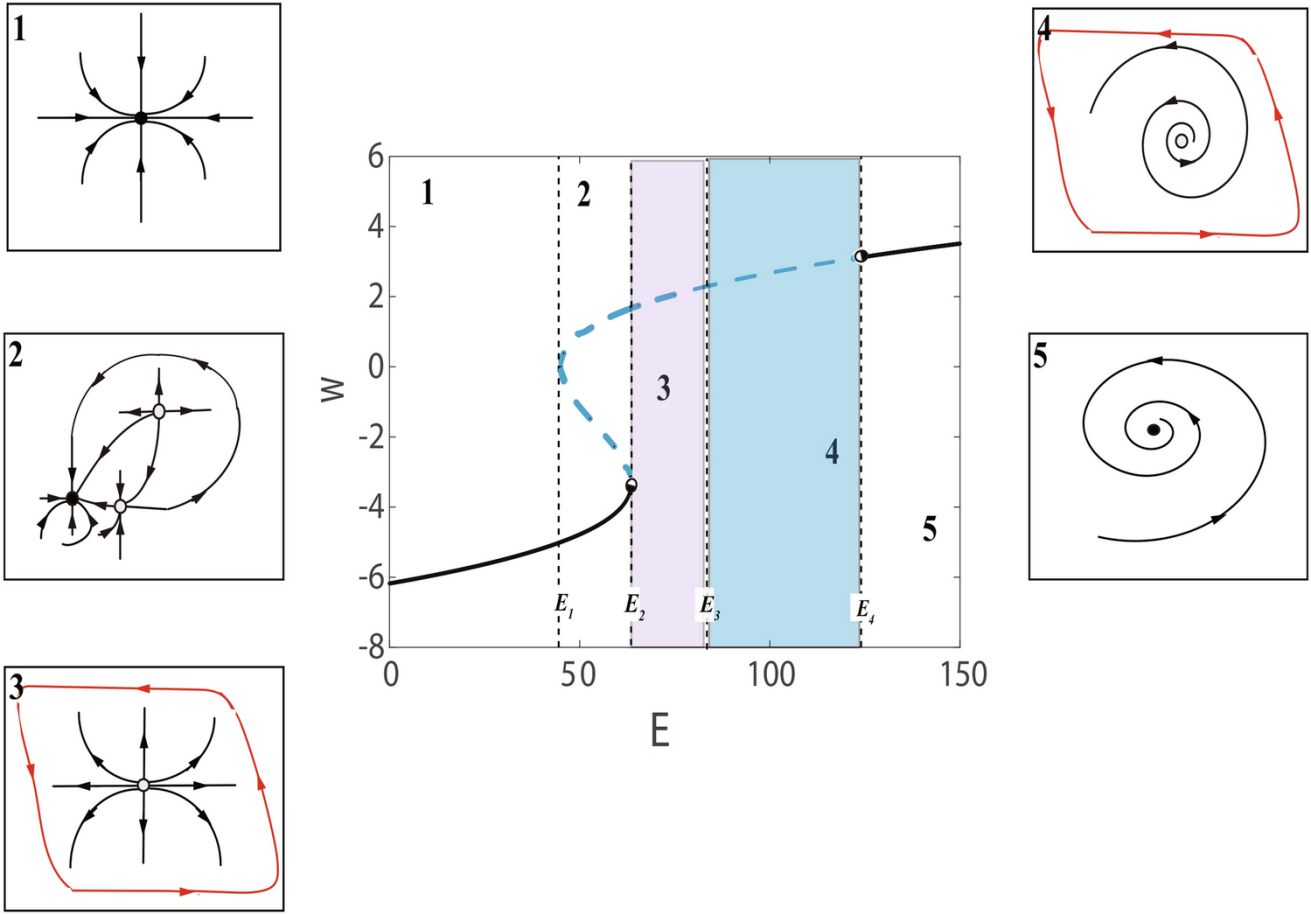
Bifurcation graph and phase portraits for the C-Q model with the parameter *E*. The black doted lines represent $E_{1}=45, E_{2}\approx 63.52, E_{3}=85.1, E_{4}\approx 123.39$. The insets shows the phase portraits of the corresponding equilibria of each interval.

The number of solutions for the parameter *E* is also analyzed in Fig. 2.

The C-Q model has one stable node during $E<45$ (as in illustration 1). In especial, $E=E_{1}=45$ or $E=E_{2}\approx 63.52$ is the critical point of the model where the model has a saddle point bifurcation. If the value of *E* gradually increases to $E_{2}$, that is, $E=E_{2}\approx 63.52$, then saddle-point appear on invariant circle bifurcations (see the illustration 2). During $45<E<63.52$, the model owns three equilibria, the stability of three equilibria (node, saddle, node) in interval ($E_{1}, E_{2}$) are respectively unstable ,unstable and stable (top-down, as in illustration 2). In interval ($E_{2}, E_{4}$), this is, $123.39>E>63.52$, one unstable point occur, and in interval ($E_{2}, E_{3}$) is unstable node (lilac, as in illustration 3), the illustration 4 shows the unstable focus (light blue) in interval ($E_{3}, E_{4}$). After $E>E_{4}=123.39$, it is stable focus. As the value of *E* gradually approaches $E_{4}\approx 123.39$ (critical point) from the direction greater than $E_{4}\approx 123.39$, once the point is reached Hopf bifurcation begins. Below we discuss these bifurcations in detail in section .

## Detailed bifurcation and dynamical behaviors

It is well known that the most important step in the qualitative analysis of any dynamical system, as well as the most advanced step, is the bifurcation analysis. Under normal conditions, the phase diagram for the model starts to change in qualitative to be related to bifurcation. For instance, as the bifurcation parameter *v* keep getting bigger, passing through the bifurcation point (saddle-point) in Fig. 1, the C-Q model is bistable, as shown in insets 2 and 3. When $v>v_{3}$, the bistability of the model disappears and another bifurcation point is passed, the stable and unstable equilibria coalesce and annihilate each other, the illustrations 4 and 5 show that one unstable fixed point appear. As the parameter *v* decreases from right to left, when $v=v_{5}$, the equilibria turns into unstable (from a stable focus to an unstable focus), Hopf bifurcation occur, as shown in insets 7 and 5. When $v<v_{3}$, the the C-Q model has two coexisting attractors.

Hence, bifurcations can decipher the emergence or disappearance for the new stable states according to the direction for motion of bifurcation parameter. Under any circumstances, the variation in the qualitative behavior for the model is related to the point of bifurcation.

**Fig. 3:**
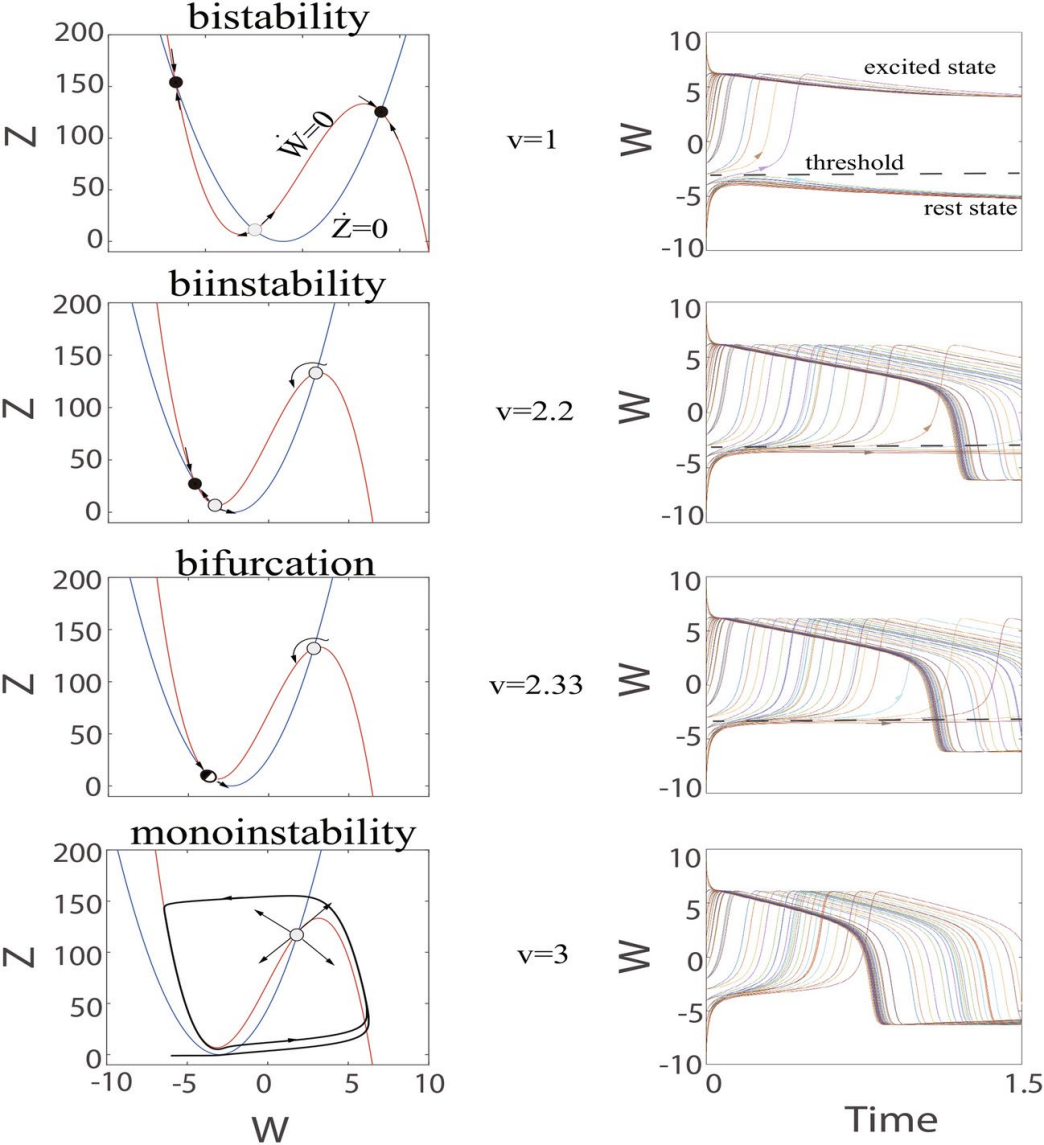
Saddle-point bifurcation in C-Q model Eq. (3). The threshold state and resting state coalesce and disappear as the parameter *v* increases.

On the other hand, initial conditions can determine whether a qualitative change in the phase portrait will manifest itself in a qualitative change in behavior. Hence, It is necessary to take into account initial conditions. We discuss the C-Q model (1) when the parameter *v* increases and describe its trajectories in Fig. 3. It is clear that the qualitative behavior of the C-Q model relies on whether the parameter *v* is less or greater than $v_{4}\thickapprox 2.3304$. As discussed above, when $v=1>v_{1}$, the C-Q model shows bistable dynamics. Some initial conditions lead to upstroke of the action potential, nevertheless others do not, in other words, the excited state and the resting state coexist (see top of Fig. 3). As *v* keeps increasing, all initial conditions result in the generation of an action potential and the resting state disappears (see bottom of Fig. 3).

Obviously, the behavior has changed qualitatively for some *v* between 1 and 3, especially $v\thickapprox 2.3304$. As the *v* gets closer to 2.3304, the distance between the stable equilibrium state and the unstable equilibrium state gradually shrinks and finally disappears. The $v\thickapprox 2.3304$ is called the bifurcation point (saddle-point) because the equilibrium points coalesce at this time. This saddle-point distinguishes two different regimes. In qualitative terms, when parameter *v* approaches 2.3304 infinitely in the direction smaller than 2.3304, the C-Q model has three equilibria points and dynamics of biinstability. Conversely, when parameter *v* approaches 2.3304 infinitely in the direction greater than 2.3304, there is a fixed point, dynamics of monoinstability and gives birth to a stable limit cycle (see bottom of Fig. 3). The exact position for the fixed point relies on the value of *v* in qualitative, however,the qualitative behavior of the model remains unchanged even if the value of *v* approaches the bifurcation point infinitely. The process in which node and saddle point approach, merge, and finally annihilate together is called saddle-point bifurcation.

**Fig. 4:**
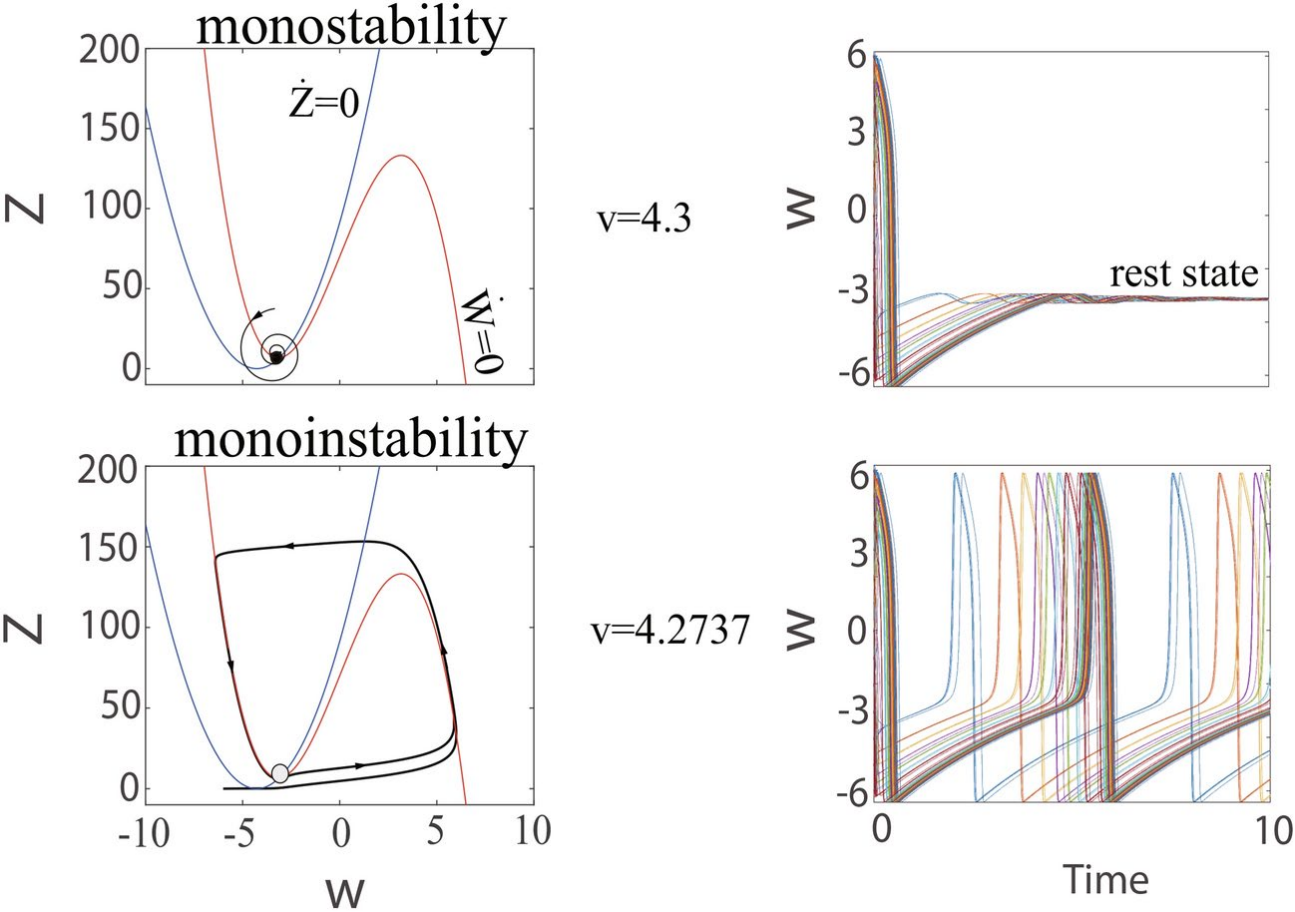
Hopf bifurcation. Transition from resting state to repetitive spiking as the parameter *v* decreases.

Subsequently, the C-Q model (1) and its trajectories when the parameter *v* decreases are analyzed in Fig. 4. One can obtain that the qualitative behavior is related to whether the value of parameter *v* is $v_{5}=4.2737$. When $v=4.3>v_{5}$, all initial conditions can not bring about upstroke of the action potential. There is only one equilibrium point and monostable dynamics (see top of Fig. 4). However, the equilibrium loses stability but does not disappear as *v* goes down to 4.2737, all initial conditions can produce action potentials. The C-Q model exhibits dynamics of monoinstability and gives birth to a stable limit cycle (see bottom of Fig. 4). The change process mentioned earlier (this is focus goes from stable to unstable) is the Hopf bifurcation. The proofs for saddle-point bifurcation, Andronov-Hopf bifurcation and the existence of limit cycle are given in “Appendix A,B and C ”, respectively.

### *Remark 1*

We also consider the C-Q model and its trajectory with different values of parameter *E* and acquire similar results. The results show that for some *E* between 40 and 124, there is a qualitative change in behavior as *E* increases, especially when $E_{2}=63.5186$, which is the saddle-point. As the parameter *E* is close to but less than 63.5186, the C-Q model has three equilibrium points and dynamics of biinstability. Otherwise, when parameter *E* approaches 63.5186 infinitely in the direction greater than 63.5186, there is a unstable equilibrium and then form a stable limit cycle. In turn, there is also a qualitative change in behavior as *E* decreases, particularly $E_{4}=123.394$. When $E=124>E_{4}$, there is one fixed point and monostable dynamics. Once $E=E_{4}=123.394$, then the fixed point becomes unstable and a stable limit cycle appears, i.e. Andronov-Hopf bifurcation.

### Bifurcation conditions

There exist one fixed point $P_{s}(-3.905,12.395)$ in C-Q model for $v\thickapprox 2.3304$. Saddle-point bifurcation occur if the following three conditions are met, it is non-hyperbolic, non-degenerate and transversal^37^.

1. The eigenvalue $\lambda _{1}, \lambda _{2}$ of the Jacobian matrix $J_s$ obtained at $P_{s}$, where $\lambda _{1}=0$, $\lambda _{2}=a\pm bi$ for $a\ne 0$.

2. The second derivative of the steady-state function with respect to *W* is nonzero, which means that the curve resembles a parabola at the bifurcation point.

10 $$\begin{aligned} \frac{1}{2}\frac{\partial f^{2}_{p}(P_{s})}{\partial W^{2}}\ne 0. \end{aligned}$$

3. $h(P_{s})$ is non-degenerate with respect to *v*.

11 $$\begin{aligned} \frac{\partial h(P_{s})}{\partial v}\ne 0. \end{aligned}$$

#### *Proof*

See “Appendix A”. $$\square$$

**Fig. 5:**
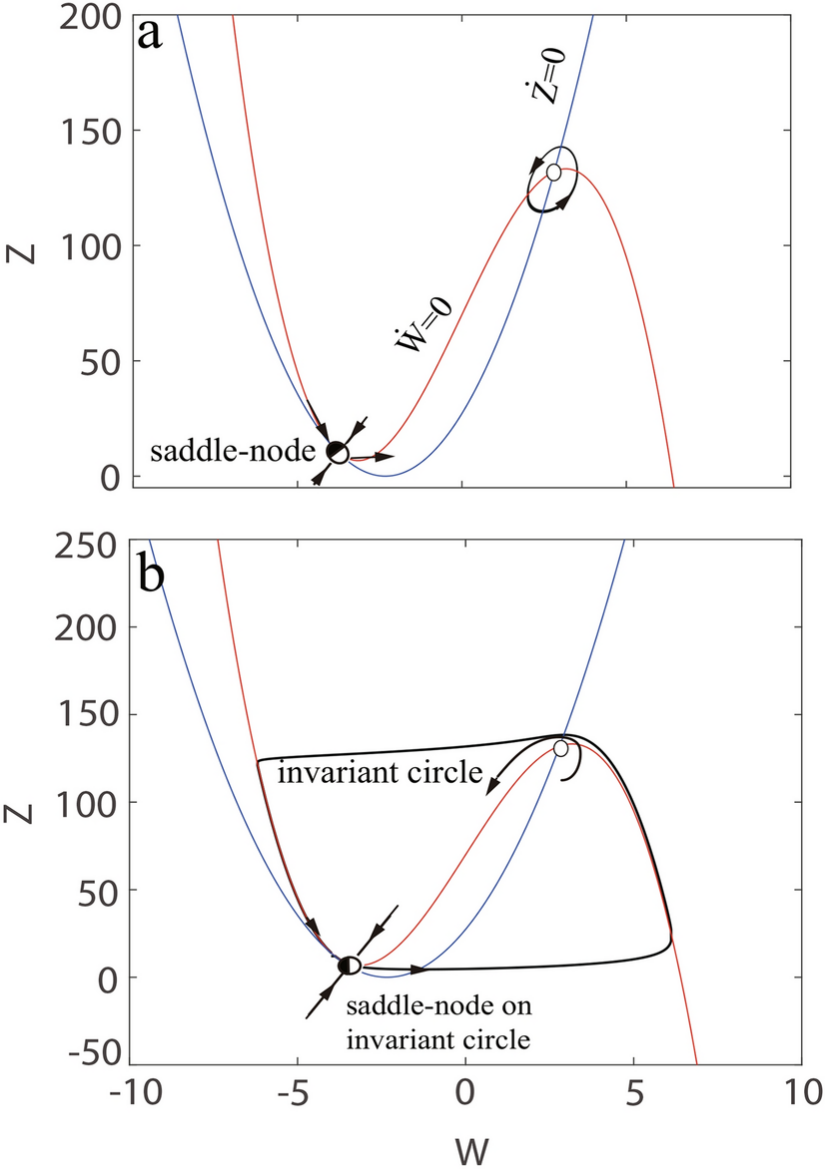
Saddle-point bifurcation with different time constant $\tau (Z)$. (**a**) Saddle-point bifurcation outside the limit cycle with $\tau (Z)=0.152$. (**b**) Saddle-point on the invariant circle bifurcation with $\tau (Z)=1$.

It is well known that saddle-point bifurcation is divided into two types: saddle-point on invariant circle bifurcation as well as saddle-point bifurcation, in especial, the saddle-point on invariant circle bifurcation is a standard saddle-point bifurcation and needs to meet certain conditions: it occurs on an invariant circle, i.e., heteroclinic trajectories or homoclinic trajectories. Both types of the bifurcation can occur in the C-Q model, as in Fig.5. The distinction between the Fig. 5b and the Fig. 5a is the time constant $\tau (Z)$ (similar to potassium ion current). This is because the time constant affects the speed of the current, on account of the speed of current is faster, it activates in time of the upstroke, thereby the magnitude of the potential goes down. And it deactivates in time of the downstroke, leading to overshoot and generating another action potential. However, when the current is slow, there is no time for deactivation in time of the downstroke, causing the undershoot and keeping resting state.

**Fig. 6:**
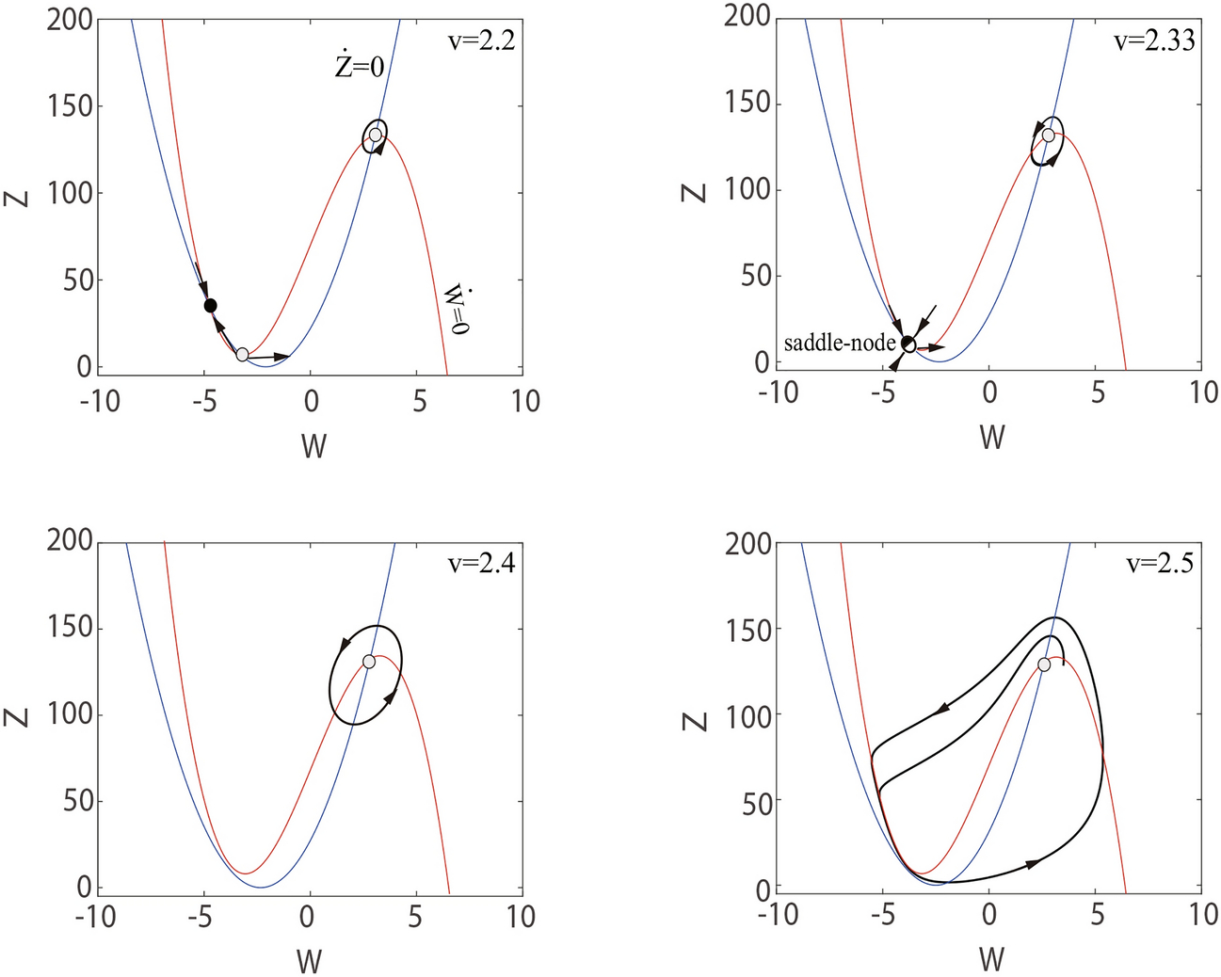
Saddle-point bifurcation with fast current $\tau (Z)=0.152$: As the value of *v* keeps getting bigger, the amplitude of the limit cycle becomes larger.

#### *Remark 2*

From the Fig. 3, when $v=2.2$, the C-Q model has two unstable equilibriums and the emergence of action potentials under some initial conditions, which seems to indicate the possibility of limit cycles. We then adjusted the time constants $\tau (Z)=6.6$ and initial conditions (near the upper right unstable focus) to obtain results similar to Fig. 10a. When the value of *v* keeps getting bigger, we find that the magnitude of the limit cycle is getting larger and larger, as shown in Fig. 6. The transition is called the saddle-point bifurcation outside the limit cycle.

The C-Q model (1) has only a equilibrium point $P_{h}(-3.109,6.781)$ for $v=4.274$. Andronov-Hopf bifurcation appear if non-hyperbolic, non-degenerate and transversal are met.

#### *Proof*

See “Appendix B”. $$\square$$

#### *Remark 3*

It is not difficult to see that saddle-point and Hopf bifurcations also occur as bifurcation parameter becomes *E*. For instance, as the bifurcation parameter *E* increases from left to right in Fig. 2, when $E>E_{1}$, the model demonstrates two unstable states, as shown in illustration 2. the unstable equilibrium point and the stable equilibrium point at $E=E_{2}$ coalesce and then annihilate together, The illustrations 3 and 4 display that one unstable fixed point appears. As the parameter *E* decreases from right to left, when $E=E_{4}$, fixed point begins to become unstable, Hopf bifurcation occur, as shown in insets 5 and 4. The C-Q model (1) not only exhibits the saddle-point on invariant circle bifurcation when the bifurcation parameter $E=63.5186$ and equilibria $P_{s}(-3.3333,0.5555)$, but also during $E\approx 123.39$ and $P_{h}(3.1091,186.6055)$, the model exhibits the Hopf bifurcation.

### Limit cycle

We can conclude that the C-Q model will exhibit the saddle-point bifurcation at $E\approx 63.5186$ or $v\approx 2.3304$ and Hopf bifurcation at $E\approx 123.39$ or $v\approx 4.274$ through the above analysis. The occurrence of these bifurcations will be accompanied by the appearance or disappearance of limit cycles, hinging on the orientation of bifurcation parameters. Such as, Fig. 10 display the left-to-right evolution, the saddle-point on invariant circle bifurcation is a bifurcation from the equilibrium point to the limit cycle. Now we reverse the direction, the limit cycle transforms into two points: node and saddle, i.e. bifurcation. Next, we select the bifurcation parameter $v=3$ and $E=70$ to prove the existence of the limit cycle.

The C-Q model (1) has an equilibrium *P*(1.903, 120.198) when the bifurcation parameter $E=70,v=3$. A stable limit cycle will appear if the following three conditions are satisfied:

1. $\exists$ a closed area *O*, where *P* is unstable and sole equilibrium in *O*.

2. the trajectory for the model can only go from the exterior to interior of the boundary of *O*.

3. The trajectory of the system does not overlap the boundary of *O*.

#### *Proof*

See “Appendix C”. $$\square$$

## Spikes, bursts and chaos of a single neuron

**Fig. 7:**
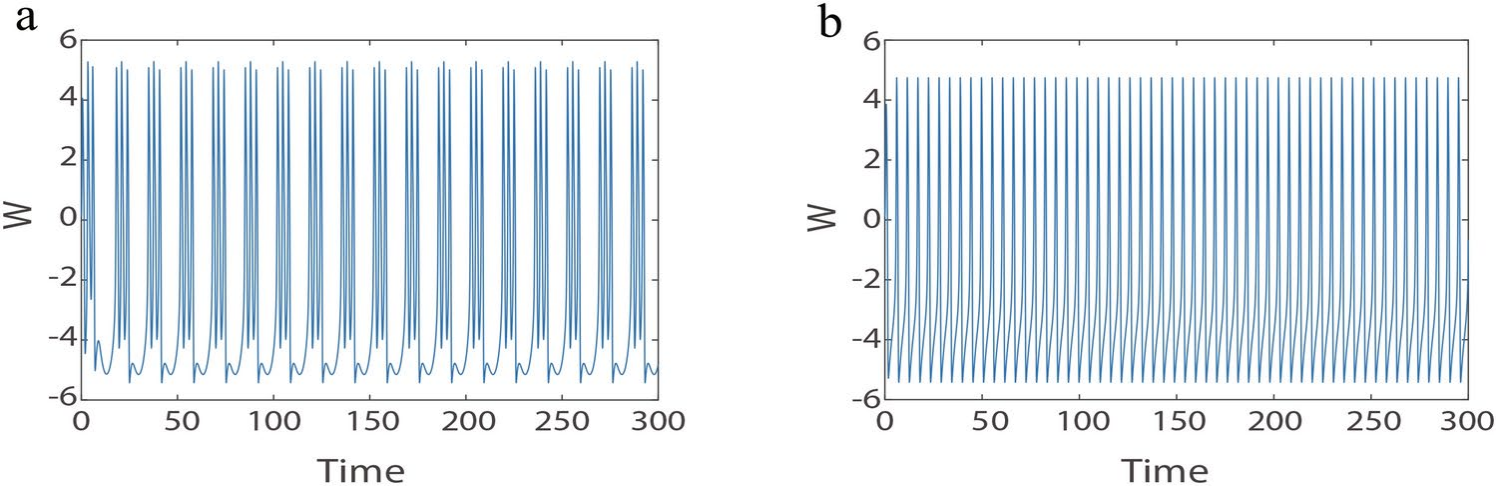
Membrane potential of the C-Q model with external input *E*. (a) Sleep stage $\omega =1.56$. (b) Wake stage $\omega =5.75$.

We learned in the previous section that the stable limit cycle is the global attractor of the system. On this basis, we try to add external inputs to gain further insight into the dynamical behaviors of C-Q model, we use the example of sleep-wake to explore the dynamics of the C-Q model. In this case, the reduced model perform functions similar to a single neuron. Neural oscillations are fundamental to achieving sleep, and many biological functions depend on sleep, hence lots of researchers have conducted extensive research on the mechanisms that neural oscillations during sleep^3,6,41^. For instance, the authors in^41^ investigated that thalamic neurons exhibit distinct spiking activities during wake and sleep under the control of the hypothalamus. During sleep, the TH neurons produce bursts, whereas spikes during wake. In this study, we design the external input current *E* to simulate the function of the hypothalamus so that the C-Q model simulates wake and sleep. In order to facilitate parameter adjustment, we perform translation scaling transformation on *Z* without changing the dynamics of model,

12 $$\begin{aligned} \frac{dW}{dt}= & -W^{3}+30W-10Z+E, \nonumber \\ \frac{dZ}{dt}= & 5(W+3)^{2}-10Z-70, \end{aligned}$$

where the external input current is devises as $E=Fsin(\omega t)+70$, which is similar to the hypothalamus. Parameters $F, \omega$ can be regulated on the basic of the actual situation. Let’s fix $F=10$, the different spiking regimes of an individual neuron achieved by adjusting the parameter $\omega$. The computational simulation are demonstrated in Fig. 7. The result show that burst during sleep, and spike during wake. The calculated results are similar to the experimental results^41^ in qualitative terms.

**Fig. 8:**
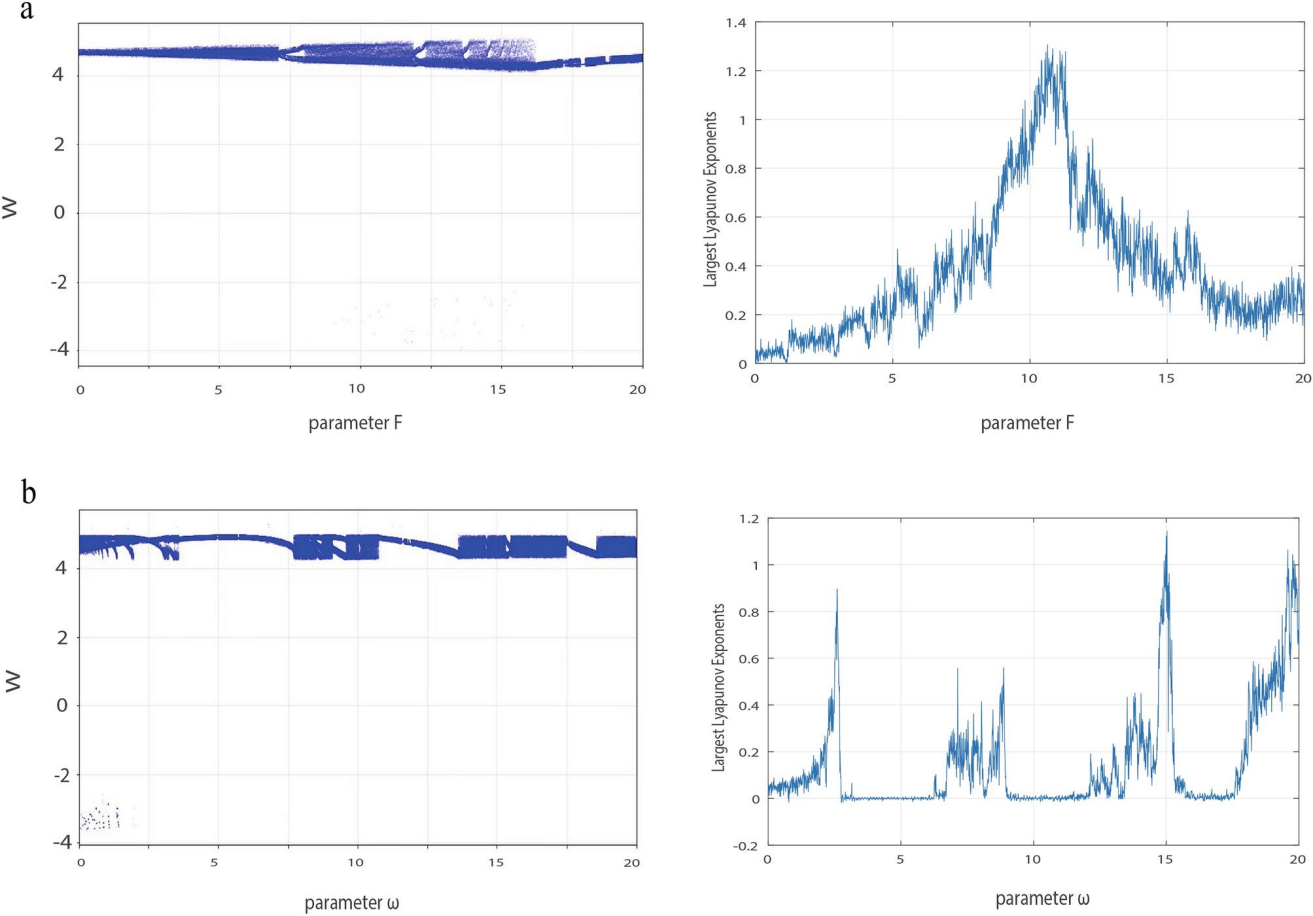
Chaos appears in the C-Q model. (**a**) Largest Lyapunov exponents and its corresponding bifurcation diagram for parameter *F* at $\omega =15.02$. (**b**) Largest Lyapunov exponents and its corresponding bifurcation diagram for parameter $\omega$ at $F=10$.

**Fig. 9:**
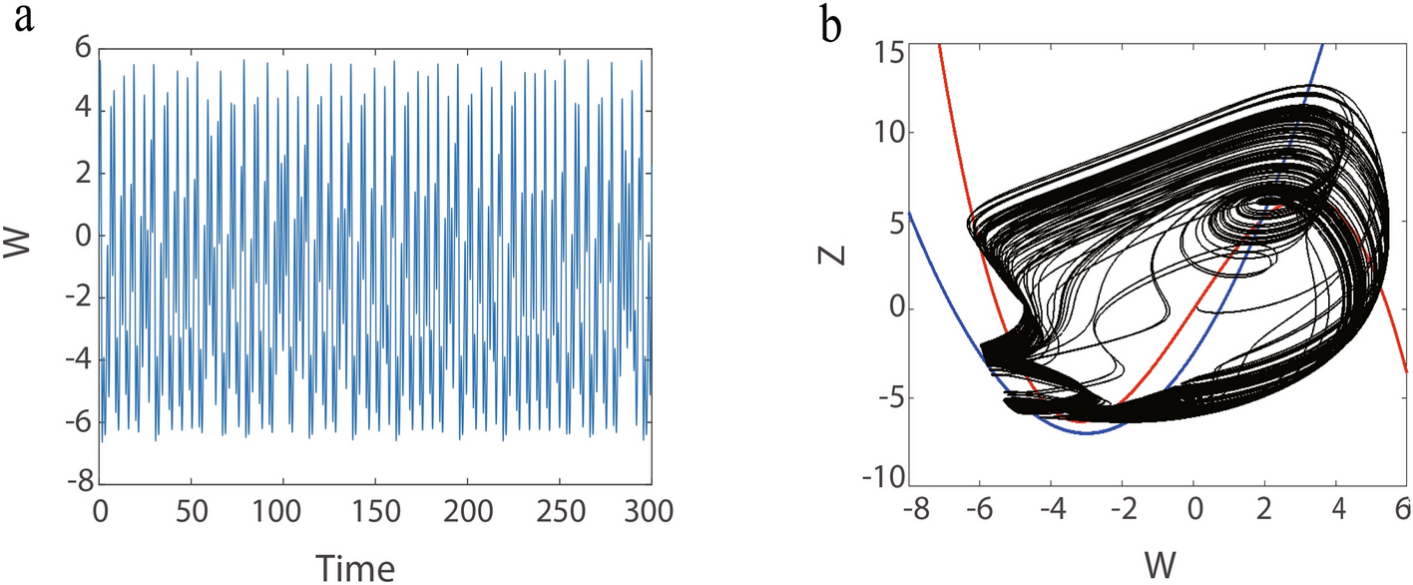
(**a**) Time series diagram at $F=10, \omega =15.02$. (**b**) Phase orbit diagram at $F=10, \omega =15.02$.

On the other hand, we keep increasing the value of $\omega$ and an interesting chaotic oscillation is observed. To verify that chaos does occur in the C-Q model. The largest Lyapunov exponents and its corresponding bifurcation diagram for the $F, \omega$ is calculated in Fig. 8. Figure 8a shows the bifurcation diagram for parameter *F* in the range 0 to 20 (step size is 0.01) and its corresponding Largest Lyapunov exponents when $\omega =15.02$. Similarly, Fig. 8b shows the bifurcation diagram for the parameter $\omega$ and its corresponding Largest Lyapunov exponents when $F=10$. These results show that chaos can emerge by adjusting *F* or $\omega$. In addition, according to Fig. 8, we select $F=10$ and $\omega =15.02$, the time series diagrams and phase portrait are given in Fig. 9.

To sum up, we can make the C-Q model appear different spiking patterns by adjusting just one parameter in the external input current *E*. In other words, at the same amplitude *F*, the spiking patterns changes as the frequency $\omega$ changes, such as spikes, bursts, chaos. Similarly, at the same frequency $\omega$, the spiking patterns can also change with the amplitude *F*.

## Conclusion and discussions

A simple and effective model, C-Q model, was proposed that can reproduce the abundant dynamical behaviors of neurons. We studied the C-Q model theoretically and numerically in different dynamical regimes. Our goal is to fully grasp the theoretical properties of the C-Q model by discussing the fixed points and their stability, bifurcation behavior and the existence of limit cycles in detail so as to better analyze the different behavior patterns of neurons. Furthermore, the C-Q model has rich dynamic behavior because we can see that not only the value of the bifurcation parameters but also the direction of the bifurcation parameters can qualitatively change the dynamic behavior of the model from the bifurcation diagram. we also observe an interesting phenomenon that we make two types of bifurcation occur in the C-Q model by tuning the time constants $\tau (Z)$ and initial conditions. Finally, a periodic input current is designed to regulate the model to simulate the basic firing mode of a single neuron, and we find that changing the frequency of the external input can produce different dynamical behaviors such as spikes, bursts, and chaos. Hence, by the control of parameters, different periodic solutions can be obtained or no periodic solutions can be obtained, thereby affecting or blocking the information transmission between neurons. These rich dynamical behaviors further provide a solid theoretical basis for analyzing the collective dynamics of brain. Meanwhile, the dynamics of the C-Q model can further provide a more solid theoretical foundation for the development of life sciences and medicine.

The obtained results may be helpful for some neurodynamic diseases, such as epilepsy. It has been reported that the EEG of epileptic brain shows discharge behavior before and after epileptic seizure, which is similar to the dynamics of C-Q model. Perhaps the C-Q model can be used to reproduce epilepsy so as to provide an idea for the treatment of epilepsy. On the other hand, the neural avalanche can be further studied by the C-Q model. The subcritical, critical and supercritical states of neural avalanche can be determined according to the diffusion coefficient and different networks, and the avalanche size, time and space distribution can be observed.

## Figures and Tables

**Fig. 10:**
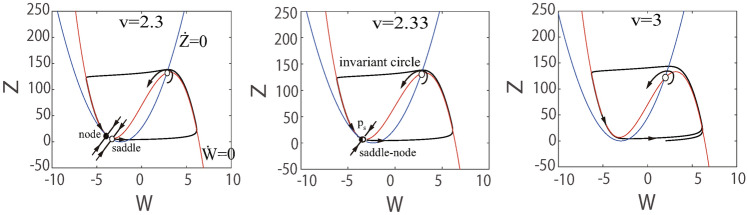
Saddle-point on invariant circle bifurcation

**Fig. 11:**
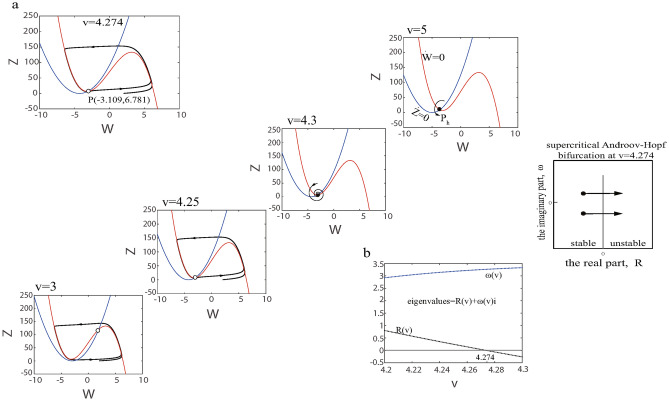
Hopf bifurcation. (**a**) As the value *v* goes down, the $P_{h}$ becomes instable and a stable limit cycle appears at $v=4.274$. (**b**) Dots represent numerical simulation of the full model

**Fig. 12:**
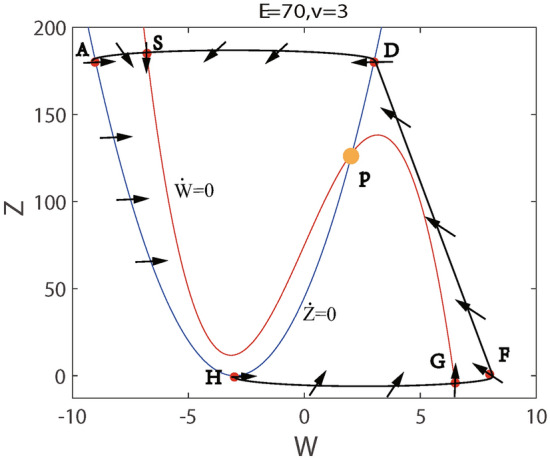
Proof of limit cycle. $O_{v}=ASDFGA$ is bold black curve and the trajectory direction of the system on *O* *v* is indicated by many black arrows. The yellow dot *P* is equilibrium

## Data Availability

All data generated or analysed during this study are included in this published article.

## Appendix A: Proof of Saddle-point Bifurcation

We prove that the saddle-point bifurcation occur when the C-Q model has one equilibrium point $P_{s}(-3.905,12.395)$ for $v\thickapprox 2.3304$.

### *Proof*

Figure 10 shows the saddle-point bifurcation occurs at $v\thickapprox 2.3304$. With the merging of saddle point and node, a heteroclinic trajectory turns into a homoclinic invariant circle. When the point disappears, the circular trajectory turns into a limit cycle.

On the grounds of the equilibrium state function, the equilibrium $P_{s}=(-3.905,12.395)$ is acquired. Then, we get

$$\begin{aligned} J_s=\left[ \begin{array}{ccc} -15.745 & ~~~~ -1\\ -15.745 & ~~~~ -1 \end{array}\right] . \end{aligned}$$

The eigenvalues $\lambda _{1}=-16.745$, $\lambda _{2}=0$ are computed.

Next, the steady-state function can be provided

$$h(W,v)=-W^{3}-5W^{2}+(30-10v)W+70-5v^{2}.$$

It is not hard to receive the following results:

$$\begin{aligned} \frac{1}{2}(\frac{\partial ^{2}h(W,v)}{\partial W^{2}})=6.71\ne 0. \end{aligned}$$

and

$$\begin{aligned} \frac{\partial h(W,v)}{\partial v}=15.74\ne 0, \end{aligned}$$

so the second and third conditions are also satisfied. Hence, the C-Q model exhibits the saddle-point bifurcation.

$$\square$$

## Appendix B: Proof of Supercritical Andronov-Hopf

Similarly, Andronov-Hopf bifurcation appear when the C-Q model has one equilibrium point $P_{h}(-3.109,6.781)$ for $v=4.274$.

### *Proof*

Similar to the proof of saddle-point bifurcation, we observe that the bifurcation appears at $v=4.274$ and $P_{h}=(-3.109,6.781)$ in Fig. 11a.

we have

$$\begin{aligned} \left[ \begin{array}{ccc} 1 & ~~~~ -1\\ 11.646 & ~~~~ -1 \end{array}\right] , \end{aligned}$$

the $\lambda _{1}=3.263i, \lambda _{2}=-3.263i$ are solved, so the non-hyperbolicity condition is satisfied.

Then, in the light of the Fig. 11b, the eigenvalues at the $P_{h}$ and bifurcation point $v=4.274$ can be approximated by

$$R(v)+\omega (v)i \approx -0.01\{v-4.274\}\pm (3.263+0.04\{v-4.274\})i.$$

Since the slope of *R*(*v*) is nonzero, the transversality condition is also satisfied. Next, we take $F_{Z}Z=-F_{W}w-\omega z$ and $W=w$, a new equation for (*w*, *z*) is obtained,

1 $$\begin{aligned} \dot{w}= & -\omega z+f(w,z), \nonumber \\ \dot{z}= & \omega w+g(w,z), \end{aligned}$$

then

2 $$\begin{aligned} f(w,z)= & 2w^{3}+70, \nonumber \\ g(w,z)= & \frac{1}{\omega }[6w^{5}-57w^{3}+(215-3\omega z)w^{2}+(10v-30)w+5v^{2}+29\omega z-2100]\nonumber \\&~&-\omega w, \end{aligned}$$

where $$v=4.274$$.

It is known in the literature^37^ that a two-dimensional system is non-degenerate, when the following formula is not zero.

3 $$\begin{aligned}&\frac{1}{16}(f_{www}+f_{wzz}+g_{wzz}+g_{zzz})+\frac{1}{16\omega }[f_{wz}(f_{ww}+f_{zz})-g_{wz}(g_{ww}+g_{zz}) \nonumber \\&-f_{ww}g_{ww}+f_{zz}g_{zz}]\ne 0. \end{aligned}$$

Based on the equation (2), that’s going to be $-245.61\ne 0$, so the non-degeneracy is also met.

Therefore, the C-Q model undergoes an Andronov-Hopf bifurcation and supercritical type. $\square$

## Appendix C: Proof of Limit cycles

Let’s fix $E=70, v=3$ to prove the limit cycle.

### *Proof*

The closed area $O_{v}=ASDFGA$ is constructed around the fixed point *P* in Fig. 12.

The $5(W+3)^{2}-Z=0$ (blue) on behalf of horizontal isoclines, the $-W^{3}+30W-Z+E=0$ (red) on behalf of vertical isocline.

A circular arc of radius 6 with a center of (-3,180) is painted, the semicircle intersects the upper left corner and upper right corner for the parabola at point A and D, and the top left corner of red curve $\dot{W}$ at point S. The semicircle equation is $Z_{ASD}=\sqrt{36-(W+3)^{2}}+180$.

The tangent direction of $Z_{ASD}$ is compared with the trajectory direction of the C-Q model. During $W\in (-9, -6.7496)$,

$$\begin{aligned} \left\{ \begin{array}{ccc} \frac{dW}{dt}& =& -W^{3}-\sqrt{-W^2-6W+27}+30W-105>0,\\ \frac{dZ}{dt}& =& -\sqrt{-W^2-6W+27}+5W^2+30W-135<0, \end{array} \right. \end{aligned}$$

During $$W\in (-6.7496, 3)$$,

$$\begin{aligned} \left\{ \begin{array}{ccc} \frac{dW}{dt}& =& 30W-\sqrt{-W^2-6W+27}-105-W^{3}<0, \\ \frac{dZ}{dt}& =& -\sqrt{-W^2-6W+27}+5W^2+30W-135<0, \end{array} \right. \end{aligned}$$

Then, the line DF $Z_{DF}=-36W-288$ is painted, which intersects the W-axis at point F, one get

$$\begin{aligned} \left\{ \begin{aligned} \frac{dW}{dt}&=-W^{3}+66W+363<0, \\ \frac{dZ}{dt}&=5(W+3)^{2}+36W+288>0 \end{aligned} \right. \end{aligned}$$

where $W\in (3,8)$, then, a circular arc of radius 5.5 with a center of (2.5, 0) is painted. The semicircle intersects the lowest point of the parabola at point H, the lower right corner of the cubic curve intersects at point G, whose trajectory is $Z_{FGH}=-\sqrt{30.25-(W-2.5)^{2}}$, we obtain

$$\begin{aligned} \left\{ \begin{aligned} \frac{dX}{dt}&=-W^{3}+30W+\sqrt{30.25-(W-2.5)^{2}}+75<0, \\ \frac{dZ}{dt}&=5(W+3)^{2}+\sqrt{30.25-(W-2.5)^{2}}>0, \end{aligned} \right. \end{aligned}$$

where $$W\in (6.4517, 8)$$.

$$\begin{aligned} \left\{ \begin{aligned} \frac{dW}{dt}&=-W^{3}+30W+\sqrt{30.25-(W-2.5)^{2}}+75>0, \\ \frac{dZ}{dt}&=5(W+3)^{2}+\sqrt{30.25-(W-2.5)^{2}}>0, \end{aligned}\right. \end{aligned}$$

where $W\in (-3, 6.4517)$. Finally, the curve HA is $Z_{HA}=5(W+3)^{2}$, during $W\in (-9, -3)$,

$$\begin{aligned} \left\{ \begin{aligned} \frac{dW}{dt}&=-W^{3}-5W^{2}+30>0, \\ \frac{dZ}{dt}&=0, \end{aligned} \right. \end{aligned}$$

Next, we discuss the orientation of special points as follows: The first point is $A(-9,180)$, $\frac{dW_{A}}{dt}>0,\frac{dZ_{A}}{dt}=0$. The second is *D*(3, 180), $\frac{dW_{D}}{dt}<0,\frac{dZ_{D}}{dt}=0$. The third is $H(-3,0)$, $\frac{dW_{H}}{dt}>0,\frac{dZ_{H}}{dt}=0$.

Hence, the trajectory for the model is from the exterior to interior of $O_{v}$. Obviously, when $E=70, v=3$, the C-Q model has at least one stable limit cycle in the area bounded by the closed curve $O_{v}$. Moreover, if *v* is determined, the fixed point is also determined and unparalleled. So the limit cycle is unparalleled by reason of the limit cycle is stable. It is easy to conclude that stable limit cycle is able to be gained in interval (2.3304, 4.2737) at $E=70$, Homoplastically, in interval (63.52, 123.39), stable limit cycle is able to be come into being at $v=3$. $\square$
